# Advances in Optical Detection of Human-Associated Pathogenic Bacteria

**DOI:** 10.3390/molecules25225256

**Published:** 2020-11-11

**Authors:** Andrea Locke, Sean Fitzgerald, Anita Mahadevan-Jansen

**Affiliations:** 1 Vanderbilt Biophotonics Center, Nashville, TN 37232, USA; andrea.locke@vanderbilt.edu (A.L.); sean.t.fitzgerald@vanderbilt.edu (S.F.); 2 Department of Biomedical Engineering, Vanderbilt University, Nashville, TN 37232, USA

**Keywords:** optical detection, Raman, infrared, fluorescence, OCT, bacterial infection

## Abstract

Bacterial infection is a global burden that results in numerous hospital visits and deaths annually. The rise of multi-drug resistant bacteria has dramatically increased this burden. Therefore, there is a clinical need to detect and identify bacteria rapidly and accurately in their native state or a culture-free environment. Current diagnostic techniques lack speed and effectiveness in detecting bacteria that are culture-negative, as well as options for in vivo detection. The optical detection of bacteria offers the potential to overcome these obstacles by providing various platforms that can detect bacteria rapidly, with minimum sample preparation, and, in some cases, culture-free directly from patient fluids or even in vivo. These modalities include infrared, Raman, and fluorescence spectroscopy, along with optical coherence tomography, interference, polarization, and laser speckle. However, these techniques are not without their own set of limitations. This review summarizes the strengths and weaknesses of utilizing each of these optical tools for rapid bacteria detection and identification.

## 1. Introduction

Bacterial and viral infections account for ~70% of all pathogenic diseases in humans [1]. Bacterial pathogens can be acquired from food, water, animals, or even clinical environments including hospitals and other healthcare settings. Once inside their host, these bacteria can exist in two general life forms: planktonic (i.e., free-floating independent cells) or in aggregates (i.e., biofilms). Most bacteria are deemed harmless until they multiply and accumulate in various regions within the body, which can then lead to the development of infection. The immune system is then triggered as an acute infection develops and the host’s immune response tries to neutralize the threat. In several cases, bacteria can evade the immune system, and the condition progresses into a persistent and chronic state requiring external interventions. Treatment with antibiotics is typically used as a remedy for the problem. However, the emergence of bacterial strains with antibiotic resistance is on the rise and is a growing global challenge. According to the Center for Disease Control and Prevention, in the United States, ~2.8 million people present infections with associated anti-microbial resistance annually [2]. This leads to prolonged treatment, extended hospital stays, and increased mortality associated with bacterial infections [3]. Though this resistance may be largely due to the overuse of antibiotics, it is also believed that certain bacteria (i.e., persistent bacteria) have innate characteristics and phenotypes that allow them to evade their host from the start of an infection. Bacteria found in biofilms and those that have adapted to intracellular growth are common examples of species that may cause persistent infections [4]. This becomes problematic when trying to identify and characterize the type of infection using gold-standard tools, which have only been tested on known susceptible strains, before administering the appropriate treatment. Therefore, there is a need for technologies that can effectively identify these microbes and their mutated strains—both in their planktonic and biofilm form—to treat patients in a timely manner and reduce healthcare and patient burden.

This review examines current challenges in the clinical detection of bacterial pathogens using conventional methods, and highlights the potential for different optical modalities to facilitate the expediated detection and identification of bacterial species causing infection.

## 2. Conventional Detection Methods

Historically, the identification of infectious pathogens was accomplished by the visual inspection of bacterial cell morphological features through a microscope. While this approach was rapid and simple, interpretation was subjective and its diagnostic accuracy was low. Currently, culture-based and molecular methods are the clinical standards for bacterial detection due to their exceptional sensitivity and specificity. However, culture-based methods are limited because cultivating bacterial colonies is labor-intensive and time-consuming. Some clinically relevant pathogens can take up to five days to grow adequate cultures [5]. Rapid pathogen identification is especially important in clinical microbiology. Faster diagnosis of the cause of infection minimizes patient risk and allows physicians to expedite treatment by prescribing pathogen-specific antibiotics rather than broad-spectrum drugs. Additionally, most microbes cannot be cultivated in standard laboratory settings because they are culture-negative [6,7]. This points to the need to develop culture-independent approaches. Alternatively, DNA-amplification techniques like polymerase chain reaction (PCR) and immunological assays like ELISA reduce detection time by eliminating the need for culturing but are complex and use costly reagents. They work on the principle that short segments of a bacteria’s DNA or antigens present on that bacteria can be used to determine its identity with a high specificity [8]. Prior knowledge about the target bacteria’s genetics or interaction with antigen-specific antibody binding is required for developing detection protocols. Therefore, each of these strategies needs specialized equipment and trained personnel to operate, but, more importantly, they are sensitive to contamination and experimental conditions [9]. Furthermore, these methods do not provide knowledge about the microenvironment, the bacterial colony morphology in these environments, or their spatial distributions. Because of these limitations, there is a growing need for new bacterial detection modalities for clinical applications that are fast, simple to operate, robust, and require minimal sample preparation.

## 3. The Case for Optical Detection of Pathogenic Bacteria

The implementation of optical methods for bacterial detection has garnered significant interest due to their quantitative nature and detection speed. While traditional techniques use the visual inspection of bacterial morphology or complex experimental protocols to identify the present strain, optical strategies are simpler in that they rely solely on the interaction of light with the bacteria. Thus, they are less subject to interpretation error or experimental variability. Various optical interactions, such as absorption, scattering, polarization, interference, and fluorescence, are accessible to probe pathogen identities [10]. Some optical techniques probe differences in cell morphology, biochemical composition, and motility to detect and/or distinguish between bacterial species and strains, while others assess the structural presence of bacterial biofilms.

For example, one of the most common ways to differentiate bacteria is their cell wall structure through Gram-status staining. Gram-positive bacteria are those that exhibit a multilayered peptidoglycan membrane containing teichoic acid. Gram-positive bacteria also present low levels of lipids and lipoproteins and high levels of murein content with no presence of lipopolysaccharides. Gram-negative bacteria present a single layer of peptidoglycan with no teichoic acids and high levels of lipids and lipoproteins due to the presence of an outer membrane. This outer membrane is composed of lipopolysaccharide complexes and is noted to be responsible for the ability of Gram-negative bacteria to be more resistant to antimicrobial interventions. Vibrational spectroscopic tools—FTIR and Raman-based spectroscopy—highlight these differences by generating spectra containing multiple peaks related to the biomolecular makeup of these cell membrane structures. Meanwhile, fluorescence allows us to conjugate sensing chemistry to the cell walls for both quantitative and visual detection. Besides cell wall morphology, bacteria can also be differentiated based on their shape (bacilli, spirals, or cocci) and motility (flagellar, spirochetal, or gliding). Techniques that use polarization and speckle contrast can analyze these characteristics through their effects on bacterial colony formations. On the other hand, bacterial biofilms are more complexed and difficult to examine in their native environment using conventional methods. Recent developments in light-based endoscopic and handheld designs offer the potential for the in vivo characterization of biofilm structures using modalities like optical coherence tomography. The study and detection of bacterial biofilms is important because biofilm-associated infections account for up to 65% of microbial infections and 80% of chronic infections in humans [11,12]. Bacteria can colonize and form biofilms in various regions of the body [13] such as the middle-ear [14,15,16], oral cavity [17,18], nasal cavity [19], tonsils [20,21], lungs of cystic fibrosis patients [22,23], heart and skin wounds [24,25], and the gastrointestinal tract [26].

Another primary benefit of an optical detection scheme is that light-matter interactions are inherently fast. The detection speed is only limited by signal acquisition parameters and post-processing computation time. However, these techniques have their own limitations. While some optical modalities such as fluorescence require simplistic device components, they require specialized chemical tags to detect the presence of pathogens. Vibrational spectroscopic techniques, on the other hand, are more flexible in their identification capabilities but involve the use of expensive photodetectors and complex system designs to achieve high sensitivity and specificity. Probing optical interactions is an indirect approach to determine a pathogen’s identity, meaning that the measured signal needs to be interpreted. The appropriate integration of statistical analysis techniques is then essential to improve the accuracy of these methods for the detection and identification of bacteria. Therefore, optical techniques can be well-suited to solve healthcare challenges relating to bacteria detection by providing fast assessment of the microbial environment with simpler operation than gold-standard techniques, but they demonstrate challenges that must be addressed to facilitate a transition into the clinical space.

Herein, this work explores the potential of different optical modalities to address current clinical challenges. These methods can provide the in situ, rapid, or real-time detection of bacterial species, strains, and mutations, as well as the distribution of bacterial biofilms in vivo.

### 3.1. Vibrational Spectroscopy to Distinguish Cell Biomolecular Composition

Vibrational spectroscopy techniques can identify bacteria by optically quantifying inherent differences in cellular biochemical content without prior knowledge of strain genetics or specialized reagents that are required in standard identification methods like PCR. Additionally, the bacterial biomass needed for acquiring reliable spectra is much less than that needed for traditional molecular-based identification approaches. When used on cultured microbe colonies, culture and analysis can be conducted within six-to-eight hours, whereas conventional detection methods require larger inoculums that can take days of culturing to achieve [27,28]. This gives vibrational spectroscopic techniques a speed advantage over current clinical practices, as the shorter incubation time needed for adequate colony growth accelerates detection time. Spectra are also acquired directly from the source pathogen. This eliminates the complex experimental protocols required for genomic assays, making identification procedures simple and sample preparation steps less laborious. Spectroscopic techniques hold the potential to be culture-independent, circumventing the need to isolate and cultivate infectious bacteria from the host in order to determine their identity. Furthermore, because this technique examines the bacteria’s biomolecular features, changes in these features may be used to pinpoint strain mutation and, thus, determine antibiotic resistance status.

**Figure 1:**
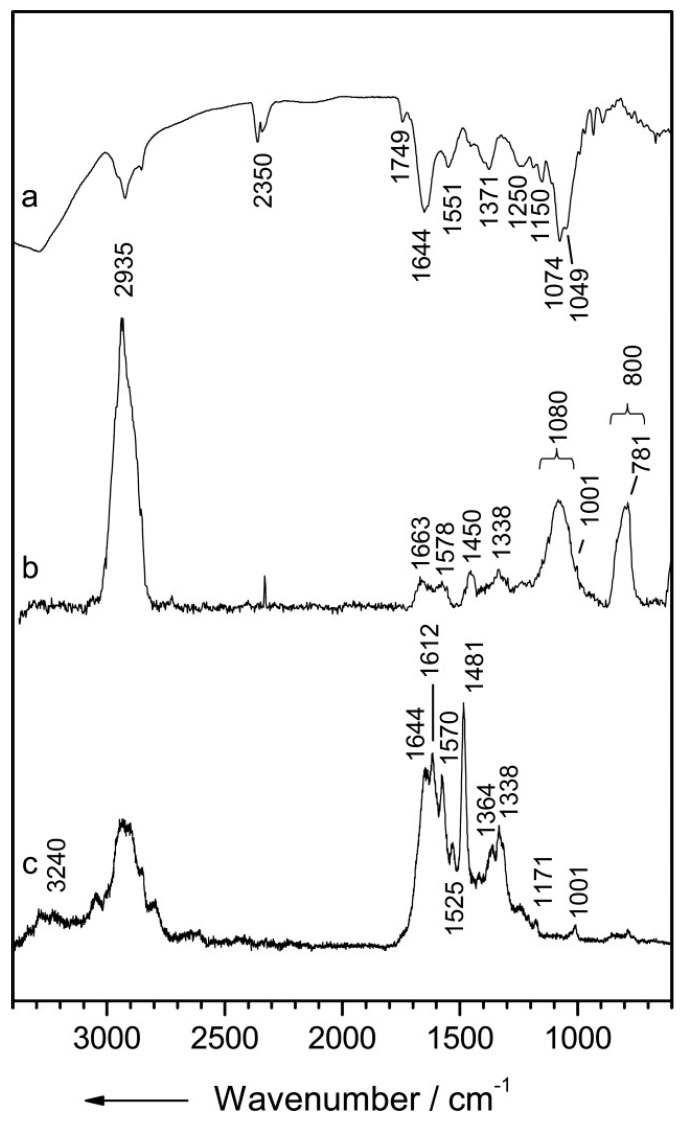
Vibrational spectra of *Streptomyces pseudovenezuela* (**a**) IR absorption spectrum, (**b**) micro-Raman spectrum with an excitation wavelength of 532 nm, and (**c**) UV-resonance Raman spectrum with an excitation wavelength of 244 nm. Reprinted with permission from [29]. Copyright © 2020, published by the International Society for Advancement of Cytometry.

The two main vibrational optical modalities are IR and Raman spectroscopy. The working principle of these techniques is that the molecular configuration of biochemicals present in the sample can be probed by their interaction with an electric field. The specific arrangement of electronic bonds within a particular molecule allows certain vibrational modes to be supported. Photons can couple with these bond vibrations through absorption or scattering events. When a photon’s energy matches the energy difference between two vibrational energy states, absorption occurs. Vibrational energy bandgaps for biological molecules typically correspond to photon energies within the mid-IR portion of the electromagnetic spectrum. The basis for IR spectroscopy is that the absorption of specific wavelengths from a broadband IR source produces a spectrum that encodes information about the analyte biomolecular composition (Figure 1a) [29]. In Raman spectroscopy (RS), the interaction between the photon and molecular bond results in an instantaneous transfer of energy between the two. This inelastic scattering event results in a change in energy of the incident photon and, therefore, a measurable shift in wavelength. The wavelength shift is similarly based on specific Raman-active vibrational modes supported by the molecules within the sample and results in its unique Raman signature (Figure 1b,c) [29].

In either type of spectroscopic technique, these vibrational excitations allow us to measure the spectrum of light that is scattered or transmitted through a sample to characterize biomolecular content and structural information. They generate a “spectral fingerprint” that is a comprehensive chemical description of the analyte at a molecular level, which can be used to characterize and identify the biological system [30]. Spectroscopic bacterial detection methods excel in their speed and simplicity of measurement. However, these techniques typically require sophisticated optical systems that utilize spectrographs or interferometers, sensitive optical detectors, and mathematical modeling techniques to perform the classification of the high-dimensional spectral information recorded. Within the last two decades, advances in laser and detector technologies, as well as chemometric analytical tools, have made vibrational spectroscopy an appealing option for the rapid, label-free, and high-throughput screening of a wide range of microorganisms [31]. The potential of these vibrational spectroscopic techniques for the clinical identification of bacteria was demonstrated by Maquelin et al., where a prospective clinical study built a reference spectral library of both IR and Raman spectra from 15 common causative agents of bloodborne infections. This library was used to develop statistical identification models that were then able to classify IR and Raman spectra from microcultures isolated from patient blood samples with 98% and 92% accuracies, respectively [27]. Since then, a large body of work has been published to explore the identification of a variety of pathogenic taxa using IR and Raman spectroscopy, and this body of work is summarized in the tables below.

#### 3.1.1. Infrared Spectroscopy

IR spectroscopy is a powerful analytical tool for the rapid characterization of molecular content and chemical structure. While earlier forms of this technique utilized dispersive gratings and a monochromator to resolve the IR absorption spectrum, this approach suffered from poor sensitivity and slow scan speeds. These have since been replaced by FTIR spectrometers, which instead use a Michelson interferometer to record an interferogram that can then be Fourier-transformed to return the IR spectrum. FTIR spectrometers are the current standard in IR spectral detection, as they have superior signal-to-noise, speed, and wavenumber accuracy compared to monochromator-based IR instruments [32]. Further advancements of this technology have led to FTIR microscopic imaging and attenuated total reflectance (ATR)-FTIR. In FTIR imaging, the incident IR beam can be focused by optical elements and scanned across the sample to provide spatially resolved spectral information and the investigation of planktonic microbial cells [33]. The integration of focal plane array (FPA) detectors has furthered the development of FTIR for clinical applications. These devices are comprised of a 2D array of pixels constructed out of IR-sensitive materials like mercury cadmium telluride, deuterated triglycine sulphate, and indium antimonide. They offer the ability to acquire thousands of IR spectra simultaneously and provide great homogeneity in the signal-to-noise (SNR) of spectra captured within the field of view of the system [34]. In ATR mode, the sample is placed onto a crystal, or internal reflection element (IRE), whose refractive index is higher than that of the analyte so that the IR beam continuously reflects off the crystal boundaries. Repeated reflections generate evanescent waves that interact with molecular components near the surface of the crystal and amplify the FTIR signal while decreasing spectral contributions of components within the surrounding media. ATR-FTIR is well-suited for the analysis of bacterial biofilms, as these thin structures can be grown on the IRE surface and selectively probed through ATR evanescent wave coupling [35].

Table 1 summarizes the body of work relating to FTIR for bacterial detection, highlighting the potential for the FTIR identification of Gram status, species, and strain, as well as biofilm characterization. Furthermore, research efforts using this tool have led to the development of extensive bacterial spectral databases like those offered by the International Journal of Systematic and Evolutionary Microbiology, which serve as a repository for further developing IR spectral classification schemes [36]. All the presented in vitro studies used reference strains or patient isolates as microbial sources due to the limitation of water absorption effects evident in biological measurements of FTIR spectra. Samples need to be dried to avoid this background noise from water bands. While some evidence points towards the FTIR detection of pathogen contamination on medical equipment using hollow-core fibers, this kind of miniaturized FTIR system is only possible due to the substrate being inherently free from the impact of overwhelming biological IR background [37]. Still, advancements in FTIR instrumentation and sample preparation methodology hold this spectroscopic technique as an active area of research for microbe detection and identification.

#### 3.1.2. Raman Spectroscopy

For every 10^6^ elastically scattered photons, approximately one photon is inelastically (Raman) scattered. Raman spectroscopy provides complementary information to IR spectroscopy about sample molecular vibrations and, therefore, both share the capacity to identify bacteria by their unique spectral biomolecular/chemical signatures. However, IR spectroscopy presents certain challenges that impede its use as a clinical diagnostic tool. Because of the limited quantum efficiency of silicon chips to IR light, this technique requires more expensive IR detectors that typically operate at cryogenic temperatures (i.e., cooled in liquid nitrogen) to reduce thermal noise. Wet samples contain an overwhelming background from the strong IR water band, requiring samples to be dried prior to measurements [66,67]. RS is advantageous for probing biological samples because the water band contribution is relatively low in the “fingerprint” spectral region, which is rich in information related to biological molecules. This simplifies sample preparation procedures and even allows spectra to be collected in aqueous solution. Another advantage of RS for spectroscopic bacteria detection is that Raman scattering can occur at any wavelength. This allows for greater design freedom to tailor excitation wavelengths, detectors, and in-line optical components to meet the needs of biological Raman spectral acquisition. The use of visible wavelengths for Raman excitation allows for the integration of RS into a standard optical microscope. These shorter wavelengths also enable higher lateral and axial resolution (<1 um), allowing for identification from smaller sample volumes and even single cells. Like FTIR, most studies utilizing RS for bacterial detection have relied on the micro-spectroscopic identification of reference strains or clinical isolates [68,69,70,71]. The ability of Raman micro-spectroscopy to probe bacterial cells in liquid suspension has drawn significant interest in the culture-free identification of bacteria directly from patient fluids. For example, Kloß et al. pioneered two studies to develop isolation protocols involving filtration and centrifugation to extract bacteria from patient sputum [72] and urine [73] samples measured by Raman micro-spectroscopy to identify the causative bacterial strains. In some cases, the biofluid spectral influence may be small enough to allow for the probing of spectral information directly from the patient sample. This has been explored in detecting pathogens from cerebrospinal fluid [74] and sputum [75] without any sample preparation steps needed. Table 2 summarizes the variety of studies using RS for bacterial identification.

The adoption of RS for bedside pathogen identification depends on system miniaturization, portability, and cost reduction. An advantage of using visible and near-infrared (NIR) wavelengths in RS is that the excitation laser and Raman emission can be coupled into cheap optical probes composed of a bundle of light delivery and collection fibers. The flexibility provided by fiber-optic light transport makes point-of-care and in-situ measurements feasible in virtually any environment. These systems typically employ smaller form-factor spectrometers to add to the portability and cost-effectiveness of their design. Since fiber optics lack spatial sampling and limit light-collection efficiency and since more miniaturized spectrometers suffer from lower spectral resolution, this implementation of RS is termed as low-resolution Raman spectroscopy (LRRS). Howell et al. first demonstrated that spectral differences were detectable from cultures of 10 clinically important bacterial species using an LRRS system [76]. Since then, these systems have been investigated by many groups for bacterial identification [77,78,79]. In vivo applications of LRRS are limited by biological Raman backgrounds from surrounding tissue or biofluid aqueous components. The inability of LRRS to spatially sample spectral information makes it difficult to separate the bacterial Raman profile from that of the background. Prior to acquiring measurements, the pathogens need to be extracted and concentrated by filtration or centrifugation. Though this adds complexity to system design and sample preparation steps, advancements in LRRS systems represent the translational potential of RS into portable, low-cost variants. While the primary challenge for the RS identification of pathogens relates to obtaining adequate SNR, Ho et al. recently showed that an artificial neural network (ANN) trained on a large enough spectral dataset could achieve identification with a 99.7% accuracy when tested on low-SNR bacterial spectra [80]. This demonstrates that improvements in chemometric tools may pave the way towards culture-free RS for classifying bacterial strain and antibiotic susceptibility.

One of the main limitations in utilizing RS is that the weak nature of Raman scattering limits the sensitivity of detection, as it must compete with other optical phenomena like autofluorescence and absorption [81]. This means that collecting vibrational spectra through the spontaneous production of Raman photons requires extremely sensitive detection hardware, long exposure times, and relatively high excitation power compared to other optical techniques. In recent years, advancements in RS methodologies such as resonance Raman and, most commonly, surface-enhanced Raman spectroscopy has allowed for significantly enhanced SNR at a much lower power. This has led to the development of commercially-available, portable RS systems, with less expensive optics, that can be utilized in clinical and remote settings.

#### 3.1.3. Ultra-Violet Resonance Raman

A resonant effect is observed when the energy of incident photons is near an electronic transition energy of a molecule. This phenomenon is called resonance Raman, which enhances the production of Raman photons by a factor of 10^3^–10^5^ for molecules meeting the criterion of having electronic transitions near the energy of the excitation source. UV-resonance Raman (UVRR) specify applications wherein the chosen excitation wavelength falls within the deep UV range (190–260 nm). Therefore, UVRR can improve upon conventional RS by preferentially enhancing Raman scattering of certain biomolecules that are important for pathogen differentiation. For instance, excitation between 222 and 257 nm has been shown to produce a selective enhancement of Raman bands associated with aromatic amino acids and nucleic acids [107,108]. An additional benefit to UVRR is that autofluorescence is absent when excited with this wavelength region, eliminating the primary source of background from the Raman spectra. This effect has been exploited for the detection and discrimination of bacteria [107]. The utility of this Raman-based modality is summarized in Table 2. While the enhancement provided by UV excitation is of clear benefit to RS, the damaging effects of high energy radiation on biological tissue are well known. This, along with the high cost and complexity of UV detection systems, limit the potential for the clinical adoption of UVRR.

#### 3.1.4. Surface-Enhanced Raman Spectroscopy

**Figure 2:**
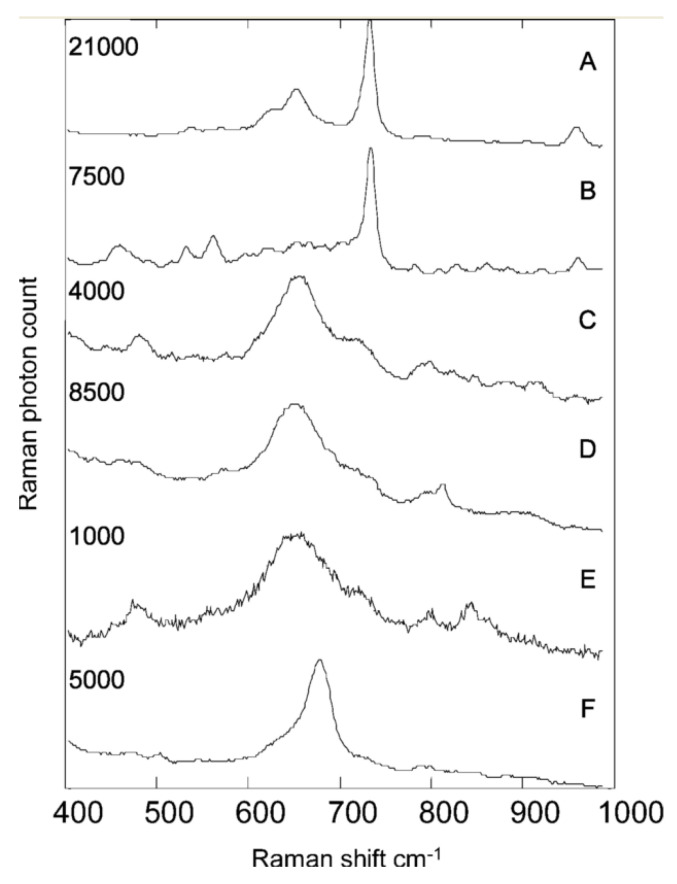
Typical unprocessed SERS spectra showing examples from UTI isolates. Each spectrum took 10 s to collect. (**A**) *Enterococcus spp.*; (**B**) *P. mirabilis*; (**C**) *E. coli*; (**D**) *K. pneumoniae*; (**E**) *K. oxytoca*; (**F**) *C. freundii*. The maximum Raman photon count for each spectrum is given on the left. Counts on the order of thousands and tens of thousands clearly indicate that these spectra result from the SERS process rather than normal Raman scattering. Reprinted with permission from [109], Copyright © 2004 American Chemical Society.

Another Raman-based modality developed to amplify the intrinsic Raman signal is surface-enhanced Raman spectroscopy (SERS). This modality achieves enhanced Raman signals without the damaging photo-degradation effect of UVRR (Figure 2) [109]. SERS utilizes colloidal nanoparticles or metallic substrates, with roughen surfaces, to dramatically increase the intrinsic Raman signal of an absorbed molecule, in close proximity, by 10^6^–10^8^ [110,111]. This enhancement is achieved by two main mechanisms—electromagnetic and chemical charge transfer. Electromagnetic enhancement generates the most significant amplification and is produced when the incident field excites surface-plasmons within the metallic structures. On the other hand, chemical enhancement is known to generate up to 10^2^ orders of magnitude amplification by transferring electrons between the nanostructures and the absorbed, chemically bound molecules. The composition of these metallic structures (e.g., silver, gold, and copper), concentration, size, and shape, as well as the dielectric environment, also dictate the degree of enhancement observed. The acquisition of an SERS signal is conducted with the same spectrometer as in conventional RS, while the addition of these metallic nanostructures provides the surface-enhanced signal. Due to the significant enhancement factor offered by SERS, the power (generally <5 mW) and signal integration times required to interrogate a sample are significantly lower than that of conventional RS. This reduction in power and acquisition time has allowed for the development of less expensive, compact, and portable and handheld Raman systems. Furthermore, the enhancements attainable with SERS have allowed for single-molecule detection and the detection of very low analyte concentration, such as bacterial samples, with little to no sample preparation.

In SERS, both label-free and immunoassay-based systems have been utilized for bacteria detection and identification. Label-free SERS is an attractive Raman modality because it amplifies the intrinsic Raman signal without the need for modifications to the metallic structures. To accomplish the SERS enhancement, metallic nanostructures can be incorporated externally or internally within the bacterial cells. There are three main methods that researchers have used to obtain label-free SERS signal from bacteria: (1) the direct growth of nanostructures on or inside the bacteria, (2) the use of colloidal nanoparticles mixed with solutions of bacteria, and (3) the use of a metallic substrate.

Early studies by Efrima et al. demonstrated growth silver colloids on and within *Escherichia coli.* For external growth, the bacterial colonies were first exposed to sodium borohydride solution before resuspending in silver nitrate solution. The silver nitrate was then reduced to form colloidal clusters that are adsorbed on the bacteria surface. Conversely, internal clusters were obtained by first exposing the cells to silver nitrate solution before the introduction of sodium borohydride (internal colloids) [93]. Externally, the SERS signal provided more unique spectral features about the bacteria compared to the Raman spectra of the untreated bacteria, which provided a more generalized biological Raman spectrum [91]. Thus, the SERS spectra can allow for improved specificity and sensitivity during cell differentiation. Efrima et al. also illuminated the differences between the cell’s internal content compared to the bacterial wall composition (Figure 2). The internal spectra provided information about the different amino acids and carboxylate molecules. In contrast, the external spectra were reported to be dominated by coenzymes derivatives of flavin such as flavin adenine dinucleotide (FAD) [112]. They also noted that acquiring SERS from within the cells is difficult due to weaker signals from sparse, isolated nanoparticles versus the ideal aggregate condition, as seen with externally grown structures, that is necessary to achieve maximal signal enhancement. One means of amplifying this signal is by administering a secondary incubation of the bacteria in colloidal silver after the first impregnation. Several other groups have used this coating or impregnation method to differentiate various species of bacteria, including Gram-positive versus Gram-negative variations (Table 2). Furthermore, the dominant FAD features in the spectra can be linked to the attractiveness of silver colloid’s nucleation at specific flavin moieties. One approach to overcoming this is by pre-synthesizing the colloidal structures prior to mixing with the bacteria cells. This method has been shown to accurately differentiate bacterial spp. and different strains such as *Bacillus* species and spores [113]. Another approach for the single-cell coating of bacteria with pre-made silver (Ag) or gold (Au) nanoparticles was shown by Kahraman et al.; here, a polyelectrolyte coating was used to deposit the nanoparticles on the cells’ surfaces using a layer-by-layer approach [114]. One caveat in using this method is that polyelectrolytes also possess a Raman cross-section and thus add non-biological features to the SERS signal that need to be extracted in signal processing and analysis steps.

Though colloidal nanoparticles offer a large surface-area-to-volume ratio necessary for sensitive detection, the lack of stability of these free-floating particles can affect the SERS signal’s reproducibility, especially across different batches of colloids. To overcome this challenge, researchers have fabricated various SERS-active substrates to improve nanostructure stability. For this reason, SERS-active substrates are a common method for label-free RS bacterial detection, especially in the analysis of clinical samples. These substrates are typically made of an array of silver or gold nanostructures deposited on various substrates (e.g., zinc oxide, glass, silicon dioxide, graphene, and polymers) [115,116,117]. These substrates have recently become commercially available due to alternative SERS applications like the detection of trace analytes [118]. Clinically, SERS-active substrates have been deployed to detect a number of bacteria biomarkers associated with infections such as *Pseudomonas aeruginosa* in cystic fibrosis patients [119] and other diseases summarized in Table 2.

In addition to label-free SERS, SERS-immunoassays have been employed for the direct detection of biomarkers, such as DNA release by the cells or antibodies expressed on cell surfaces. These assays are similar to ELISA-based assays. However, pairing immunological specificity with SERS offers the ability to multiplex and detect multiple species simultaneously on one platform with a single wavelength laser. These assays can easily be incorporated within the paper and micro-fluidic devices for rapid, disposable lab-on-a-chip readings. Many of these fluidic device mechanisms can filter and isolate bacterial cells for improved specificity and sensitivity with decreased sample volumes [120].

SERS offers many advantages over conventional RS due to its increased signal strength. It can allow for the improved differentiation of species and strains without heavily relying on multivariate analysis tools for the discrimination [121,122,123]. However, caution needs to be taken when using the different forms of SERS. In label-free SERS, the signal generated may be influenced by the byproduct of laser-induced photochemical defects. Therefore, it is wise to utilize lasers with very low power (~1 mW). Additionally, due to concerns regarding the signal reproducibility and long-term stability of colloidal particles from batch to batch, multiple measurements need to be taken and averaged for best results. Batches also need to be re-assessed over long periods [124]. The use of SERS substrates over colloidal nanoparticles also significantly reduces these concerns. In terms of SERS-based immunoassays, one is limited in requiring prior knowledge of the targeted species within the samples. However, once known, this technique has proven powerful in detecting very low concentrations of biological analytes with a sensitivity beyond that of the typical ELISA assays, with a faster response time and minimum sample preparation.

In summary, vibrational spectroscopy offers unique advantages in understanding the biomolecular make-up of bacterial specimens. However, the presence of spectral signatures from all biological components makes these spectra complex and may require additional pre-processing and multivariate analysis to accurately classify samples. Therefore, in most cases, once bacterial spectra are acquired, the pre-processing of the data is done via algorithms such as polynomial fitting and rubber-band baseline correction to remove signal contributions from autofluorescence or the environmental background [125,126]. Outliers can also be removed using spectral quality metrics like SNR and principle component analysis (PCA) [127]. Next, noise filtering (e.g., smoothing) using PCA or a Savitzky-Golay (SG) filter mathematical methods [128,129] and normalization are performed [130]. After preprocessing, mathematical modeling techniques are then used to classify samples based on species, strain, or antibiotic resistance status. The most common unsupervised models include PCA, K-means, and hierarchal cluster analysis (HCA). Popular supervised models require training on a subset of spectra with class labels and include algorithms like artificial neural networks (ANNs), canonical variate analysis (CVA), and discriminate analysis (DA), amongst other regression models [131].

#### 3.1.5. Fluorescence

Fluorescence spectroscopy is widely utilized within the scientific research community and clinical settings for a variety of applications. This technique relies on the specific excitation and emission characteristics of fluorescent molecules to selectively image or quantify biomarkers related to various disease states. The basic instrumentation required to detect a fluorescence signal includes a light source to excite electronic energy states of the fluorescent molecules, emission filters to reject excitation light and other emissions not related to the molecular species being probed, and a photodetector to measure the fluorescent signal. This simplicity in instrumentation has allowed fluorescence to be applied to various applications, including bacterial detection. One of the most common forms of this modality used for bacterial detection is fluorescence in situ hybridization (FISH). FISH does not require the prior culturing of specimens and is an improvement over typical PCR because it allows for the direct visualization and identification of bacteria within its microbial environment. It involves labeling oligonucleotides with specialized fluorescent probes to detect individual microbial cells by hybridizing specific nucleic acids within an intact cell. The key to the success and accuracy of FISH relies on oligonucleotides selections, which need to be specific, sensitive, and relatively short-chained to allow for the ease of cell penetration [132]. Once inside the cell, hybridization must be carried out under stringent experimental conditions including temperature, humidity, and the right amount of light exposure (to avoid photo-bleaching) for the successful binding to a target before washing and visualizing with a fluorescent microscope.

**Figure 3:**
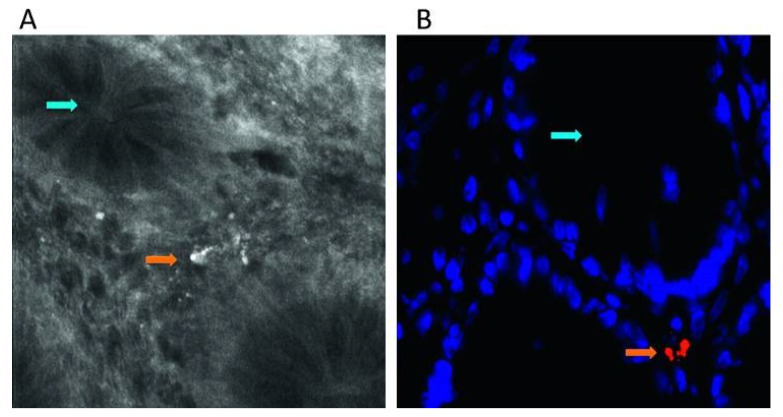
Intramucosal bacteria in human colon identified at confocal laser endomicroscopy and fluorescence in situ hybridization (FISH). (**A**) Fluorescent intramucosal bacteria within the lamina propria can readily be identified using fluorescein-aided endomicroscopy. Single crypts are shown with their characteristic round appearance (blue arrow). Single bacteria and clustered bacteria (orange arrow) can be identified within the lamina propria between two crypts (pericryptal space). (**B**) FISH testing confirmed the presence of intramucosal bacteria due to the bright red fluorescence. The nuclei and RNA are shown in blue. Reprinted with permission from [133]. Copyright © 2020, published by BMJ Publishing Group Ltd. and the British Society of Gastroenterology.

FISH has been used in detecting the inflammation of the gastrointestinal tract such as inflammatory bowel diseases, ulcerative colitis, and Crohn’s disease, where pathogenesis is linked to changes in the homeostasis of the gastrointestinal microbial environment. Researchers have looked to the application of FISH as a means to rapidly assess biopsy tissues procured from these areas to examine the bacterial population. Human colon biopsies [133,134,135] and fecal material [136,137] from patients and healthy controls have been investigated to visualize and identify the bacteria present and to quantitatively determine the percent change in bacterial concentration, as well as the location of the bacteria within the biological specimen, across different patient cohorts (Figure 3). FISH has also been used to assess biofilm-associated bacterial infections such as chronic wound infections [24,138]. The presence of bacterial biofilms in chronic wounds such as foot ulcers, pressure ulcers, and other skin-related ulcers is believed to be responsible for the inability of that wound to heal [139]. Studies using FISH have shown that the biofilms can be located either near the surface of the wound or deep down within the wound bed, depending on the species of bacteria causing infection. Therefore, this tool can be used to track the healing process of a wound, potentially in its early stage, to alter the treatment method if needed. In chronic otitis media, FISH has been used to evaluate and identify bacterial present in the biofilms of the middle-ear and upper respiratory tract mucosa extracted from patients [140,141,142,143].

Other forms of fluorescent detection utilize techniques such as fluorescence resonance energy transfer (FRET) [144,145,146], fluorescence polarization [147,148], and chemi/bioluminescence [149,150,151,152,153] to detect pathogens in multiplexed environments. These assays either utilize DNA hybridization methods or antibody-sandwich assays for binding to targeted molecules and can be coupled to different nanoparticle structures (e.g., quantum dots, silica, and gold) [154,155]. These biosensors are similar to the SERS-based immunoassays; for example, fluorescently-labelled silica particles conjugated with bacterial antibodies were designed for multiplexed detection of *E. coli*, *Salmonella typhimurium,* and *Staphylococcus aureus* in a FRET-based system [156]. The measurements can be taken in less than an hour following the introduction of the conjugated nanoparticles mixed in excess with the bacteria before washing to remove unbound particles. However, the main advantages of fluorescence over SERS are its simplicity in the optical instrumentation that allows for less expensive device designs and the ease of usage and data interpretation. Unlike SERS, fluorescence is limited in its multiplexing capabilities, as highly multiplexed systems can lead to the cross-reactivity of the labels, resulting in false positives. Therefore, this approach is typically limited to three or less fluorescent labels.

**Figure 4:**
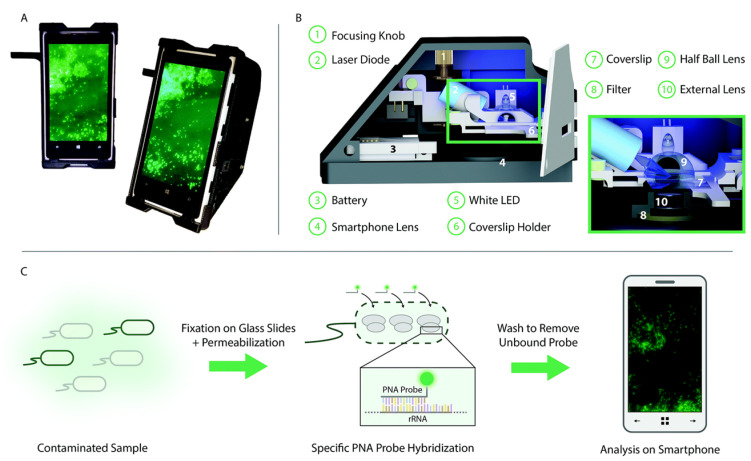
Selective targeting and imaging of single bacteria on a smartphone. (**A**) Photographs of a smartphone microscope displaying images of fluorescently labeled *Cronobacter* spp. bacteria. (**B**) 3D illustration of the same optomechanical unit that is mounted on the smartphone in (**A**). (**C**) Schematic illustration of the bacterial detection procedure. Bacteria from the contaminated sample are fixed on 22 × 50 mm^2^ glass slides, and the bacterial membrane is permeabilized in order for the peptide nucleic acid (PNA) probe to enter the bacteria. An Alexa Fluor 488 dye is chemically linked to the PNA probe that, in turn, is designed to bind specifically to certain regions of the ribosomal RNA (rRNA) of the bacteria. After washing away unbound probes, only the targeted bacteria remain fluorescent and can be imaged using the smartphone-based microscope shown in (**A**). Reprinted with permission from [168]. Published by The Royal Society of Chemistry.

Another strength of using fluorescent biosensors for the detection of bacteria is in their portability. Fluorescent biosensors have been employed within microarrays or microfluidic platforms for the rapid detection of pathogens [61,149,157,158,159,160,161]. Moreover, with the advancement of smartphones, these platforms have been coupled with mobile devices to move the detection of bacteria beyond benchtop microscopes and flow cell systems [162,163,164,165,166]. For example, Shrivastava et al. recently designed a 3D printed body attachment for a smartphone device that incorporated an objective lens, excitation and emission filters, and a white light LED illumination source for the fluorescent imaging of captured bacteria (Figure 4) [164]. This device also included a lab-on-a-chip device comprising magnetic pads to capture a fluorescently labeled aptamer assay designed to detect *S. aureus*. There is also an open-source “do it yourself” publication that allows researchers to fabricate their smartphone fluorescent microscope that can be used for pathogenic detection [167]; it plays to the strength of the simplicity in the optics and user-friendly nature of fluorescence detection.

### 3.2. Optical Coherence Tomography

Optical coherence tomography (OCT) offers a unique optical imaging approach based on light scattering to non-invasively visualize and characterize bacterial biofilms and effusions. It is a label-free technique that utilizes back-scattered NIR light to probe biological tissues and provide high-resolution cross-sectional images of tissue morphology in real-time. The image contrast is generated by differences in local scattering from various tissue layers that exhibit changes inrefractive index. Typically, the system comprises a low-coherence light source coupled into an interferometry system composed of sample and reference arms. At the sample arm, the light is focused across the specimen using lenses and scanning optics. The backscattered light from varying depths then interferes with light from the reference arm and is focused onto a photodetector. OCT is most commonly used in diseases relating to the eye, both clinically and experimentally. However, its application space has expanded in recent years due to advancements in system design and processing techniques that have shown promise for this modality to be applied to other clinical diagnostic areas, including the middle ear, skin, esophagus, and dental tissue [169].

**Figure 5:**
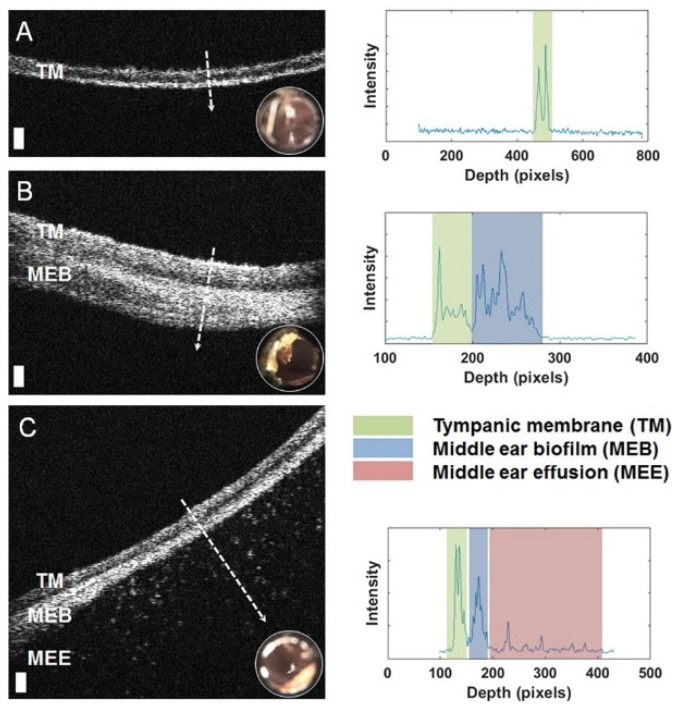
Left: Representative optical coherence tomography (OCT) cross-sectional (B-scan) images and A-line profiles. (**A**) OCT and digital otoscopy (inset) data from a normal ear. (**B**) Data from an ear with a middle ear biofilm (MEB). The A-line profile shows additional scattering behind the tympanic membrane (TM). (**C**) Subject with middle ear fluid (MEF) and an MEB. Right: The scattering profile shows three distinct regions in the scan. White dashed lines denote the location of the A-line scan within the OCT B-scan. Scale bars represent 100 micrometers in depth. Reprinted with permission from [178] Copyright © 2020, published by Springer Nature.

In relation to bacterial infections, OCT has been used for the detection and characterization of biofilm formation. The images provided by OCT offer micron-scale resolution of biofilm structure, therefore making it well-suited for tracking biofilm growth dynamics within in vitro studies due to the low power levels needed to generate these images [170,171]. Advances in OCT images analytics has expanded its capabilities to measure metrics like biofilm porosity and mechanical properties [172]. The most explored clinical application of OCT is in acute otitis media (AOM). AOM is an inflammation of the middle ear with an onset of middle ear effusion (MEE) typically caused by bacteria but sometimes caused by viruses or both. Due to the over-prescription of antibiotics and antibiotic resistance in AOM therapy, it is crucial to identify the source of infection before administering treatment. Researchers at University of Illinois Urbana-Champaign have shown the promise of a handheld OCT system for the in vivo diagnosis of the different forms of OM (e.g., AOM, otitis media with effusion (OME), and chronic OME) and to detect the presence of MEE (Figure 5) [173,174,175,176,177,178]. This system can detect the presence of biofilms of the tympanic membrane (TM) and can characterize the structure of biofilms (e.g., thickness and heterogeneity), including any presence and type of effusion fluid (i.e., serous vs. non-serous) to determine the severity of the infections. Recently, this system became commercialized as the TOMi^TM^Scope (available by PhotoniCare) and has received the Food and Drug Administration (FDA) clearance.

OCT is less commonly used in other types of biofilm-associated diseases. However, in recent years, some groups have been evaluating the use of this modality in areas such as wound healing in relation to *Staphylococcus aureus* skin infection [179], biofilm growth and effects on demineralization of the tooth enamel [17], and biofilm detection in chronic rhinosinusitis patients [180]. In all these applications, OCT images provided information on whether a biofilm was present, the thickness and scattering of this film, and the location of the film in relation to adjacent tissue layers. To validate these images, histopathology staining typically follows these measurements.

Drawbacks to using OCT for bacterial detection are the inability to characterize and identify the species of the bacteria associated with the biofilm and effusions. Therefore, for bacterial identification, this technique needs to be coupled with optical spectroscopy tools to improve the utility of this modality towards the simultaneous imaging and speciation of biofilms. One promising multimodal approach is a Raman coupled low-coherence interferometry (LCI) system where Raman can identify the type of bacteria and the LCI provides depth-resolved images of the biofilm and effusion state [181].

### 3.3. Interference

**Figure 6:**
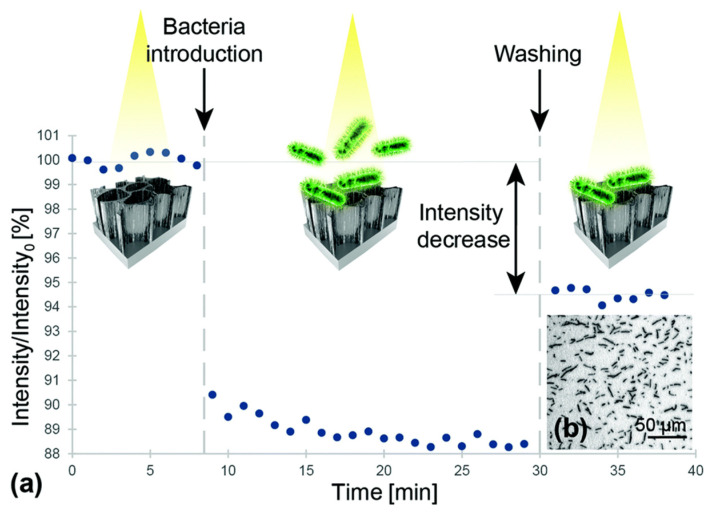
(**a**) Relative intensity change of the interference signal upon exposure to *Lactobacillus acidophilus* bacterial suspensions (10^7^ cells per mL). First, a baseline was established in buffer solution After incubation with bacteria suspension, the biosensor was extensively washed before continued signal readout Note: the intensity values were normalized to the initial average intensity, marked as intensity. (**b**) Microscope image taken immediately after the biosensing experiment depicts *L. acidophilus* cells captured onto the aptamer-modified PSiO_2_. Reprinted with permission from [183]. Published by The Royal Society of Chemistry.

While OCT systems employ interferometers to investigate biofilm morphology, alternative biosensor designs utilizing interferometers have been investigated for specific bacterial detection. These systems sacrifice imaging capabilities and instead attempt to detect the presence of planktonic bacteria through interference effects. This is accomplished by the functionalization of the sample arm with antigen-targeting agents, like aptamers or antibodies, to selectively bind specific bacterial species. Upon binding, the presence of a bacterial layer within that arm of the interferometer affects the interference signal that is measured. An early attempt to develop interference-based selective detection was developed by Massad-Ivanir et al., where a porous SiO_2_ nanostructure conjugated with a monoclonal antibody acted as a Fabry–Pérot thin film. When targeted bacteria are captured by this system, the change in thickness of this film generates a modulation in an optical signal reflected off the surface due to thin-film interference [182]. Urmann et al. designed a similar thin-film system using aptamers to target and detect *Lactobacillus acidophilus* in a mixed bacterial population (Figure 6) [183].

**Figure 7:**
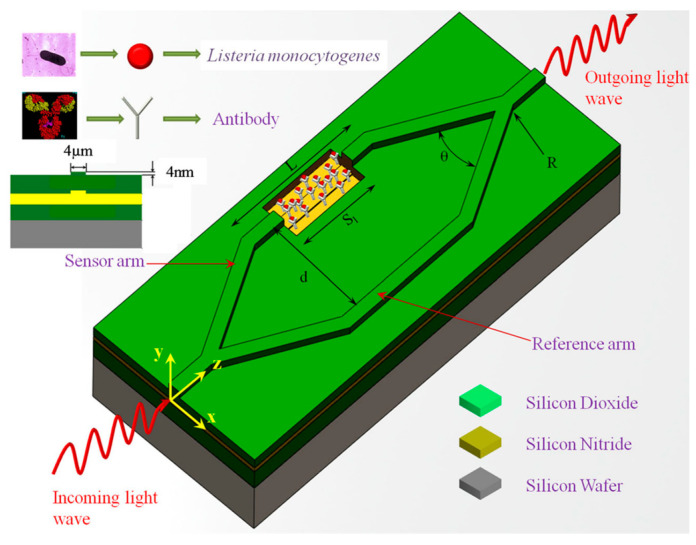
Schematic representation of biofunctionalization on Mach-Zehnder interferometer (MZI). Here, Sl is the length of the sensor area, L is the length of the sensor arm, d is the distance between the sensor and reference arms, and θ is the opening angle of the Y-divisor for angular Y-junctions, whereas R is the radius of curvature of the Y-divisor for S-bend Y-junctions. Reproduced with permission from [185]. Copyright © 2020, published by Springer Nature.

Other attempts to exploit interference effects for bacterial detection have employed interferometer systems built into waveguides. Sarkar et al. demonstrated that a Mach–Zehnder interferometer (MZI) built onto a silicon waveguide could detect *Listeria* in solution (Figure 7) [184]. A light-beam propagating through the waveguide is split into two arms that then converge and generate an interference signal. Bacteria are captured by antibodies in one arm that induces a change in local refractive index and, therefore, a phase shift of light traveling through this arm. This added phase causes a change of intensity in the interference signal and provides species-specific optical detection in a low-cost MZI biosensor. Janik et al. built an MZI directly into an optical fiber by machining a small cavity through the cladding and core [185]. This cavity was functionalized with bacterial phages to immobilize free-floating bacteria. Light traveling through the fiber was split at this cavity where a phase shift induced by captured bacteria generated a similar change in the interference signal, allowing for the detection of *E. coli.*

### 3.4. Polarization

**Figure 8:**
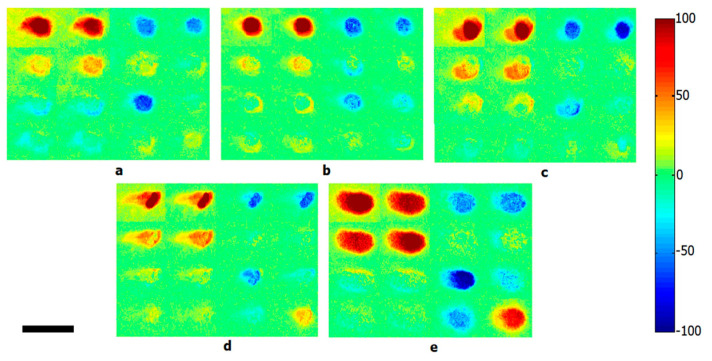
Calculated backscattering Mueller matrix images of five different bacterial colonies: (**a**) *Escherichia coli*, (**b**) *Lactobacillus rhamnosus*, (**c**) *Rhodococcus erythropolis*, (**d**) *Staphylococcus aureus*, and (**e**) bacteria-free Luria broth agar media. Scale bar is 2 mm. Reprinted with permission from [187]. Copyright © 2020, published by Springer Nature.

Polarization can also be used to assess the morphological features of bacterial species. Bacteria exhibit certain basic shapes and are categorized as such: coccus, spiral, bacillus, etc. They also display species-specific arrangements in culture, as cells divide and self-organize into distinctive colony patterns [186]. The polarization state of light is sensitive to the microstructure of biological systems and has been explored as a possible avenue for bacterial identification. In polarimetry, the Mueller matrix is a popular tool to characterize the interaction of various states of polarized light with a sample. Mueller matrix polarimetry was used to differentiate bacterial cultures by Badieyan et al., who developed a polarization-based imaging modality for species differentiation; elements of the back-calculated Mueller matrix encoded structural information about colony patterns within the culture and provided imaging contrast to discriminate between *E. coli*, *Lactobacillus rhamnosus*, *Rhodococcus erythropolis*, and *Staphylococcus aureus* (Figure 8) [187].

### 3.5. Laser Scattering

**Figure 9:**
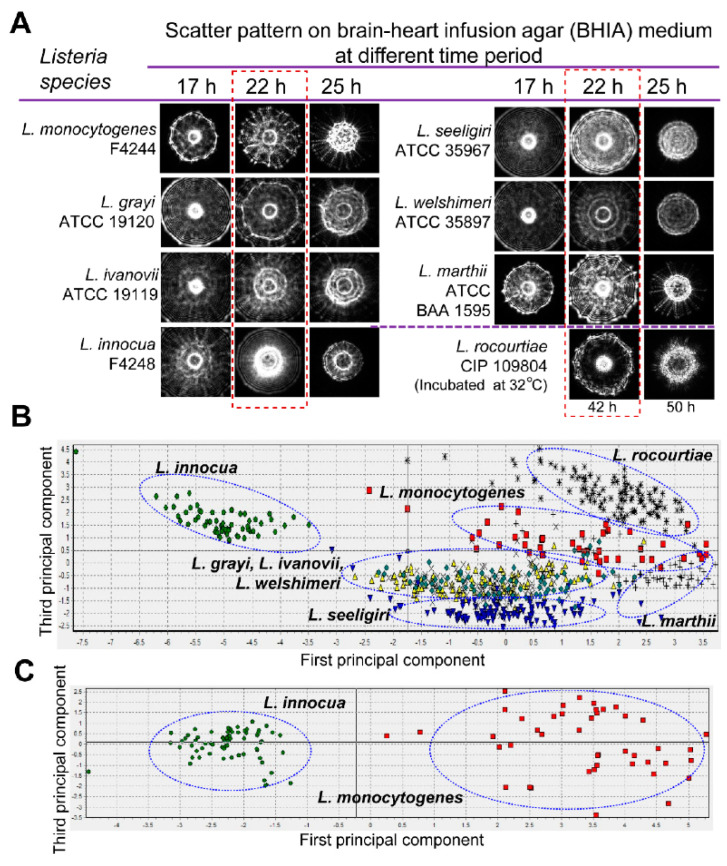
Optical scatter patterns of *Listeria* species and image analysis. (**A**) Colony scatter patterns were captured using bacterial rapid detection using optical light-scattering technology (BARDOT) at different incubation times for eight *Listeria* species on brain heart infusion agar plates. The rectangular selection with broken line depicts the optimal incubation time (22 h) that yielded differentiating scatter images when the colony size was 1.1 ± 0.2 mm diameter. (**B**) Principal component analysis of the eight *Listeria* species used to build the scatter image library. Blue oval selections indicate grouping of the *Listeria* species. (**C**) Principal component analysis of *Listeria* *monocytogenes* and *Listeria innocua* colony scatter images that were used to build a two-species scatter image library. The blue oval selections indicate the grouping of each *Listeria* species. Reprinted with permission from [191]. Published by Multidisciplinary Digital Publishing Institute.

Similar to polarization-based identification, laser scattering is sensitive to colony formation morphology. The micro-structure of a bacterial colony depends on the shape of the cells and the way that they are arrange in colonies. Therefore, the scattered light’s interference from a unique bacterial colony architecture generates a diffraction pattern, often called a scatterogram. This scatterogram is generated by exposing the colony with a coherent laser beam, which is then captured by a camera and interpreted with feature-extraction algorithms. This technique uses elastic scattering to encode information about the morphology of the sample and so is named elastic light scattering (ELS). Bayraktar et al. were the first to demonstrate bacterial identification with a simple ELS system containing only a collimated diode laser source, detector screen, and camera that was used to differentiate six *Listeria* species [188]. Forward scattered light through the bacterial colony generates a scatterogram on the screen, which is then imaged onto the camera. Statistical metrics, called moment invariants, of the collected image, were extracted to quantify spatial patterns present within the scatterogram. ANNs were then able to classify the present species using these pattern metrics with accuracies of 88–98%. Similar strategies to discriminate against various *Listeria* species were also conducted by Bae and Banada [189,190]. The simplicity of such an ELS system design for scattering-based identification led to the development of an automated detection system called bacterial rapid detection using optical light-scattering technology (BARDOT) by Advanced Bioimaging Systems (Figure 9) [191]. BARDOT has been implemented in a variety of bacterial studies to identify and distinguish various pathogenic species and strains, including *Listeria* [191,192,193], *Salmonella* [194,195], *Bacillus* [191], *Vibrio* [196], and *Campylobacter* [197].

While these studies indicated that ELS detection methodologies are capable of species differentiation through transparent media, clinical diagnostic settings often use opaque or turbid media because many pathogenic bacteria require blood-supplemented agar. This severely limits the applicability of BARDOT and other forward-scattering ELS technologies for clinical use. Genuer et al. developed an alternative system design to facilitate the measuring of the scatterogram from back-scattered light [198]. By passing a linearly polarized coherent beam through a quarter-wave plate, the now circularly polarized light interacts with a bacterial colony and scatters back towards the detection path. As it passes back through the quarter-wave plate, it again becomes linearly polarized, but it does so perpendicular to the incident beam. This allows the back-scattered light to be separated from the incident light by a polarizing beam splitter. This device, named MICRODIFF system, was shown to have an equal discriminatory ability to forward-scattered variants. While the identification of species is possible by the analyzing the unique characteristics of the scatterogram, Choi et al. performed scattering-based bacterial detection directly from urine with a microfluidic system [199]. When a urine sample is subject to an electric field applied across the microfluidic channel, free-floating bacteria within the fluid align themselves to the field orientation, thus influencing the preferential scattering angle. By placing a detector at this angular position, this group was able to detect pathogens causing a urinary tract infection present in the flow region by an increase in scattered light intensity, demonstrating a simple and label-free method to determine the presence of bacteria without any image processing.

### 3.6. Speckle Contrast Imaging

Laser speckle contrast imaging (LSCI) also takes advantage of elastic scattering but adds sensitivity to particle mobility. Scattered light from moving particles causes fluctuations in the otherwise static diffraction pattern; this is called “speckle” in the context of LSCI. These fluctuations manifest as a random, high contrast granular pattern that can be detected in the same manner as ELS systems. The dynamics of this speckle pattern give information about the degree of mobility of particles within the field of observation. Sendra et al. used LSCI to monitor the motile response of *P. aeruginosa* to tryptone, a peptide mixture that attracts bacteria in culture [200]. Towards bacterial characterization, LSCI was later used to study differences in bacterial species mobility. It was shown from experiments of *Bacillus*, a genus characterized as being highly mobile, that different species produced strikingly different LSCI speckle patterns [194]. *Bacillus polymyxa* produced static patterns consistent with other genera, while those of other *Bacillus* species were overwhelmed with speckle effects. Kim et al. exploited this finding to analyze differences in the swarming growth behaviors of *Bacillus* species using LSCI, and they found that the average speckle grain size and spatial contrast decreased while the number speckles increased during bacterial colony growth [191]. Because LSCI is low-cost, label-free, and requires very little laser power, the speckle parameters measured by this technique offer a scattering-based optical strategy for the real-time monitoring of bacterial growth kinetics over extended periods [201]. Though *Bacillus* produces the most notable speckle contrast, all bacteria are motile to some degree during colony growth, and so LSCI is capable of monitoring growth behavior across many genera. This is an extremely attractive option for antibiotic susceptibility testing, as the administration of antibiotics to culture impacts susceptible bacterial growth and induces a change in the measured speckle parameters. Han et al. showed that this approach could determine antibiotic susceptibility in *E. coli, S. aureus*, and *P. aeruginosa* in two-to-four hours, which typically takes 16–20 h using standard protocols [202].

## 4. Comparison of Optical Techniques

Of the reviewed optical strategies for bacterial detection, there is a clear distinction between techniques that perform specific strain detection and those capable of performing general species identification. Fluorescence-based detection systems, as well as interferometer biosensors, require the binding of bacteria to antigen-specific targets and represent examples of optical systems that must be designed for the detection of a particular strain. By transducing the binding of pathogens into a direct change in optical signal, they offer greater design freedom by their simplicity in signal interpretation, and therefore are well-suited for miniaturized system development that are more easily deployable. Being culture-free strategies, they can potentially capture bacteria from patient biofluids for rapid detection. Techniques like vibrational spectroscopy, polarimetry, and ELS fall into the second category because they indirectly identify bacterial species by unique features derived from their respective optical signals. FTIR/Raman spectra, Mueller matrix element values, and ELS scatterograms are analyzed to extract quantitative features that aid in species differentiation. By training classification algorithms on these features from representative datasets of isolated bacterial strains, these statistical models can then be used to classify an unknown sample’s identity. In most cases, these measurements must be conducted on isolated bacterial colonies, although advances in SERS platforms have attempted to bridge this gap. The remaining optical methods do not perform speciation, instead utilizing signal metrics to characterize bacterial growth kinetics. OCT provides a label-free measurement of the formation of biofilms that can be used to track biofilm growth for in vitro studies and the in vivo detection of infection status in diseases like otitis media. LSCI, similarly, has shown merit as a potential tool to monitor bacterial mobility for the purpose of drug sensitivity screening and clinical antibiotic susceptibility testing. A comparison of these methods, along with the explored sample types and technique accuracy or the limit of detection (LOD), is summarized in Table 3.

## 5. Future Prospective and Current Clinical Limitations

Advancements in the design of new optical systems have demonstrated the immense potential for various optical spectroscopy, imaging, and sensing tools to assess pathogens within biological samples. These techniques offer rapid, and in some cases, culture-free approaches to detect and characterize bacterial species with simplistic measurement protocols.

When aiming to study and characterize bacteria biofilms in their native environments, as in the case of OM, investigators can seek to use portable OCT systems to examine biofilm structures and, where applicable, types of bacterial effusions. Advances in its in vivo applications now involve using machine learning to automate OM classification based on the images acquired to better aid medical personnel ease in interpreting the OCT images [178]. Beyond OM utility, researchers are taking advantage of OCT imaging capabilities for other in vivo biofilm assessment relating to rhinitis, dental plaques, and antibiotic therapy monitoring [204].

If the goal is to detect and identify bacteria species, strain, or study antibiotic response, Raman spectroscopy and FTIR are the best tools to use—especially if the species is unknown. However, these modalities are best used when coupled with known databases or neural network algorithms to improve specificity. Moving towards culture-free detection and in cases where culture-negative bacteria are being investigated, SERS-based techniques can prove beneficial and may be best suited for this application. The significant amplification of the intrinsic Raman signal that the nanoparticles provide is advantageous for culture-free methods. In recent years, nanoparticles have been coupled at the tip of fiber optical probes to create a more reproducible means of acquiring the SERS signal [205]. Therefore, with improved sensitivity over conventional Raman optical probes, this technique can provide researchers further flexibility in its use, depending on the ex vivo and in vivo environment. One drawback that researchers need to be aware of is that the SERS-based optical probes and label-free assays need to be in direct contact with the sample. Further research needs to be conducted to assess the sensitivity and specificity of the optical probes in identifying a particular species without labels. If the bacterial species is known, fluorescence, SERS, and interference-based platforms can be used for direct targeting using antigen-specific binding. Of the three, fluorescence spectroscopy is the simplest and most inexpensive tool to use. It is also the most versatile because it can be used for both imaging and quantitative analysis.

To assess bacterial growth kinetics, laser speckle contrast imaging and OCT are the most useful tools. However, with simpler optics, laser speckle is more advantageous and provides faster information, especially in measuring antimicrobial susceptibility. Compared to the gold standard and commercial antimicrobial susceptibility test (AST), which can take anywhere between 8 and 20 h for standard methods, laser speckle contrast can accomplish this within two-to-five hours [202]. If interested in colony growth patterns, polarization and laser-scattering offer users the ability to capture growth patterns over time and utilize those patterns to understand how the cells divide and self-organize into colonies.

Towards point-of-care diagnostics is the miniaturization of fluorescence and SERS-based systems into a mobile or handheld platform for field applications. The simplicity in the optics required for fluorescent-based spectroscopy and imaging allows it to be fashioned into compact handheld devices that can be coupled with microfluidic platforms to be used at the point-of-care [164,166,206]. Similarly, with recent advancements in the design of SERS-based probes and compact spectrophotometers, these systems can couple with both paper- and microfluidic-based tools for point-of-care analysis. However, SERS-based systems are more expensive and have a higher degree of complexity than fluorescent ones, but they do provide the possibility for multiplexed detection.

Bacteria identification and the visualization of the distribution within a localized space can be achieved using various multimodal optical systems. These systems involve the merger of two or more optical modalities to take advantage of the benefits from the individual systems and provide users with more comprehensive information for bacteria detection and characterization. For example, coupling the biomolecular sensitivity of Raman with OCT, which provides depth-resolved imaging of a bacterial environment, can allow users to interrogate bacteria within a localized space [181]. Another multimodal system involves the coupling of autofluorescence with OCT for dental biofilm assessment. The fluorescence imaging provides information about the maturity of pathogenic plaque and OCT about the total plaque buildup [207].

However, while these optical modalities have shown tremendous promise in detecting bacteria, there are still several challenges associated with these techniques that must be addressed. The broader challenge centers around developing analytical software for quick and reliable data processing and real-time interpretation. For some modalities, such as FTIR, this is already being addressed with the creation of large databases containing information on various biological samples that are open access and can be shared and used as standards by which unknown data can be interpreted. Such databases are in their early stages, and unanimous consensus among scholars in each field is needed to ensure the validity of the information.

Another major challenge is the cost and expertise required to construct the various technologies. For fluorescence devices, this is less of an issue, as discussed. The recent acceptance and use of Raman spectroscopy in forensic science and pharmacology have allowed for the commercialization of various RS tools from benchtop to optical probes and handheld devices. This technique still relies on the use of sensitive detection hardware and narrowband laser sources, which are relatively expensive components. Still, the reduction in prices of these tools is inevitable as the market for these kinds of systems matures. In addition, as more clinicians become aware of the recent FDA-cleared OCT handheld device for otitis media detection, this technology also has the potential to become more widely available for the common use of biofilm characterization. Research studies with other technologies such as laser speckle contrast, inference, and polarization are still in nascent stages. Therefore, these systems are not readily available, as most are designed and built in-house.

The optical detection of pathogenic bacteria is currently an area of tremendous ongoing research because different modalities have the potential to provide information about the biomolecular make-up of a species, growth pattern recognition, single-cell versus biofilm characterization, cell motility and viability, cell mutation, and antibiotic resistance status. However, further research must be pursued to enable the eventual use of these modalities by clinicians.

## Figures and Tables

**Table 1:** Summary of work relating to FTIR-based systems for bacterial detection. ATR: attenuated total reflectance.

Modality	Spectral Information Used for Bacteria Classification	Sample	Limitations
FTIR	Gram-positive vs. Gram-negative [38,39] Polysaccharide: 900–1200 cm^−1^ Amide (proteins/peptides): 1500–1800 cm^−1^ Cell membrane fatty acid chains (-CH3, -CH2, -CH stretch): 2800–3000 cm^−1^	Cultured isolates (i.e., *Pseudomonas* *aeruginosa*, *Klebsiella* *pneumoniae*, *Enterobacter* *cloacae*, and *Acinetobacter* *baumannii*)	Strong water absorption requiring dried samples; Limited information via direct visual analysis; needs multivariate analysis methods to provide discrimination; Samples cultured up to 24 hr at 37 °C ideal for measurements
	Bacteria Species/Strain Differentiation [27,40,41,42,43,44,45,46,47,48,49] Polysaccharide: 900–1200 cm^−1^ Proteins/Free amino acids/Polysaccharides: 1200–1450 cm^−1^ RNA/DNA/Phospholipids: 1200–1250 cm^−1^ Proteins:1500–1700 cm^−1^ Fatty acids: 2800–3000 cm^−1^	Cultured isolates (i.e., *Pseudomonas*, *Bacillus*, *Staphylococcus*, *Candida*, *Enterococcus*, and *Streptococcus*, *Enterobacter*)	
	Antibiotics Resistance [50,51,52,53] Nucleic acid: 1200–1300 cm^−1^ Carbohydrates: 950–1200 cm^−1^	Cultured patient specimens (i.e., *Escherichia* *coli* and *Staphylococcus)*	
	Identification of Bacteria from Patient Samples [54,55,56]	Sputum (cystic fibrosis) and urine (urinary tract infections)	
ATR-FTIR	Bacterial biofilms [57,58] Polysaccharide: 950–1200 cm^−1^ Amide I: 1650 cm^−1^ Amide II: 1550 cm^−1^ Species/strain differentiation [59,60] Phospholipids/DNA/RNA: 1185–1500 cm^−1^ Carbohydrates:900–1185 cm^−1^	In vitro (i.e., *Caulobacter* and *Streptococcus*) Cultured isolates (i.e., *Acinetobacter baumannii)*	Lack of spatial sampling; ATR accessories needed for measurements; Additional processing steps required for comparable absorption spectra; Limited information via direct visual analysis; needs multivariate analysis methods to provide discrimination
FTIR-Imaging	Cultures printed in microarray and microfluidics [61,62,63,64] Differentiation of strain and Gram-positive vs. Gram-negative [65]	i.e., *Listeria, Enterobacter, Klebsiella, Escherichia, Staphylococcus, Bacillus,* and *Pseudomonas*	10 µm resolution; difficult to probe single bacterial cell; Specialized microscope slides (i.e., zinc selenide crystals) to reduce background and requires more sophisticated optical elements (i.e., FPA detectors, IR-transmissive lenses); Limited information via direct visual analysis; needs multivariate analysis methods to provide discrimination

**Table 2:** Summary of work relating to Raman-based systems for bacterial detection. SERS: surface-enhanced Raman spectroscopy.

Raman Modality	Spectral Information Used for Bacteria Classification	Sample	Limitation
Conventional RS	Bacteria species/strain differentiation [71,82,83,84] Amino/nucleic acids: 700–1100 cm^−1^ Amide I, II, III: 1640–80, 1552, and 1220–1310 cm^−1^	Cultured clinical oral *Streptococci ssp*. Cultured *Escherichiacoli* (*E. coli*) strains, *Haemophilus influenzae*/*Moraxella catarrhalis*/*Streptococcus. Pneumoniae (S. pneumoniae)*, Group B *Streptococcus*/*E. coli*, and *Staphylococcus* *aureus (S. aureus)*	Requires low Raman background microscope slides such as calcium fluoride or quartz; Limited information via direct visual analysis; needs machine learning methods to provide discrimination
	Culture-free patient samples [85,86] DNA: 788, 1093, and 1578 cm^−1^ Proteins: 1004, 1250, and 1658 cm^−1^ CH-vibrations: 1341 and 1452 cm^−1^	Urine (lab-on-a-chip device)	
	Antibiotic resistance [87] Amino acids/DNA: 765–935 cm^−1^ CH_2_/CH_3_ bending:1431–1464 cm^−1^: Carotenoids: 1159 and 1523 cm^−1^	Cultured *S. aureus* mutant strains	
UV Resonance (~244 nm)	Bacteria classification [88,89] Nucleic acids: 1475–1600 cm^−1^	Cultured urine isolates, *Bacillus* strains	Photo-degradation effect causing cell damage; Limited information via direct visual analysis; needs machine learning methods to provide discrimination
	Antibiotic resistance [90] Nucleic acid/protein ratio: 1480/1607 cm^−1^	Cultured *Bacillus pumilus*	
Surface-enhanced Raman spectroscopy (culture-free)	Nanoparticle growth external or inside cells [91,92,93,94] External (cell wall features) Amino acids, proteins, carboxylate, flavin adenine dinucleotide (FAD), lipids, and DNA Internal: Cytosolic protein (1250 cm^−1^) and nucleic acids	*E. coli*, *Pseudomonas aeruginosa*, *Methicillin-resistant Staphylococcus aureus* and *Listeria spp*., *Geobacter sulfurreducens*, and *Bacillus megaterium(B. megaterium)*	Nanoparticles need to be in close proximity (<3 nm) with cell surface; Material, shape and size dramatically affect signal strength and spectral profile
	Mixture of nanoparticles with bacteria Live vs. dead bacteria [95] Gram-positive vs. Gram-negative [96]	*E. coli* O157, *Salmonella typhimurium(S. typhimurium)*, *S. aureus*, and *B. megaterium*	
	Label-free SERS substrate Urinary tract infection [97] Bacterial meningitis [98] Human blood [99,100] Surface charge: poly-electrolyte coated magnetic nanoparticles [101]	*Lactobacillus plantarum*, *E. coli*, *S. aureus*, *Pseudomonas aeruginosa*, *Klebsiella oxytoca*, *S. pneumoniae*, *Haemophilus influenzae*	
SERS biomarker assays	SERS biomarker assays (indirect detection) Antibody assays [102,103,104] Aptamer assays [105,106]	*S. aureus*, *E. coli*, Multi-drug resistant *S. typhimurium, S. aureus*, and *E. coli*	

**Table 3:** Comparison of application, sample type and accuracy/limit of detection (LOD) for various optical modalities.

Purpose	Modality	Sample Types	Accuracy (%)/LOD
Species-specific detection via ligand binding	FISH	Biopsy tissue [133,134,135]	NA
		Fecal matter [136,137]	NA
		In vivo biofilms [24,138,140,141,142,143]	NA
	FRET	Cell suspension [144,145,146]	15–300 CFU/mL
		Blood cultures [156]	10 ng/mL
	Fluorescence polarization	Whole blood [148]	1 CFU/mL
	Fluorescence biosensor	Cell suspension [149,158,159,160,161,165,166]	10^2^–10^8^ CFU/mL
		Mixed cell suspension [163,164]	10^2^–10^3^ CFU/mL
	Interferometry	Mixed cell suspension [183,184]	10^5^–10^6^ CFU/mL
		Cell suspension/biofilm growth [185]	100 CFU/mL
Species and/or strain identification via machine learning	Vibrational spectroscopy	Clinical isolates [38,39,41,42,43,44,45,46,47,48,49,50,51,52,53,54,55,56,59,60,61,62,63,64,71,82,83,84,88,90]	75–100%
		Urine [85,86]	10^7^ CFU/mL, 100%
		Biofilms [203]	NA
	SERS biosensor	Clinical isolates [91,92,93,94,95,96,97,98,99,124]	84–100%
		Urine [97]	10^5^ CFU/mL, 97%
		Whole blood [99,100]	11 CFU/mL, 100%
		Cerebrospinal fluid [98]	NA
	Polarimetry	Bacterial cultures [187]	NA
	ELS	Bacterial cultures [188,189,190,191,192,193,194,195,196,197,198]	80–98%
		Urine [197]	10^7^ CFU/mL
Bacterial growth kinetics	OCT	In vitro biofilms [170,171,174]	NA
		In vivo biofilms [173,174,175,176,177,178,179,180,181]	NA
	LSCI	Bacterial cultures [191,194,200,201]	NA
